# Carveol-Derived Cycloaliphatic Epoxy for Bio-Based High-Performance Thermosets

**DOI:** 10.3390/polym18202531

**Published:** 2026-10-15

**Authors:** Chae Hyun Lim, Miji Kim, Seok Il Yun

**Affiliations:** Department of Chemical Engineering and Materials Science, College of Biochemical Engineering, College of Convergence Engineering, Sangmyung University, Seoul 03016, Republic of Korea; simonlch0813@naver.com (C.H.L.); zagro102@naver.com (M.K.)

**Keywords:** bio-derived epoxy, carveol, terpene, urethane crosslinking

## Abstract

Carveol, a terpenoid alcohol, was selected as a platform molecule for the development of high-performance bio-based epoxy thermosets. A cycloaliphatic diepoxy monomer, carveol dioxide (CDO), was synthesized and subsequently cured with branched polyethyleneimine (BPEI) to afford epoxy thermosets exhibiting outstanding thermomechanical properties, including a storage modulus of 2.5 GPa at 30 °C and a glass transition temperature (*T*_g_) of 97 °C. The resulting thermoset also exhibited strong adhesion to stainless steel, reaching a lap-shear strength of 10.1 MPa, which compares favorably with previously reported bio-based epoxy adhesives. To further enhance network performance, the pendant secondary hydroxyl group of CDO was exploited to introduce urethane crosslinks prior to epoxy curing. Urethane incorporation markedly increased the crosslink density and raised *T*_g_ to 137 °C, which, to the best of our knowledge, is the highest value reported for terpene-based epoxy thermosets. These results highlight carveol as a promising renewable platform for high-performance epoxy thermosets and adhesives because its unique combination of C=C bonds and a secondary hydroxyl group enables both epoxide formation and subsequent network modification.

## 1. Introduction

The depletion of fossil resources and growing environmental concerns have accelerated the development of biomass-derived polymers as sustainable alternatives to conventional petroleum-based materials [1,2,3]. Among thermosetting polymers, epoxy resins are particularly important, because their highly strained oxirane rings readily react with a wide variety of hardeners, forming highly crosslinked networks with excellent mechanical properties, high glass transition temperatures (*T*_g_), outstanding chemical and corrosion resistance, strong adhesion, low cure shrinkage, and excellent electrical insulation properties [4,5,6,7]. However, the vast majority of commercially available epoxy resins are still derived from petroleum-based feedstocks. In particular, approximately 90% of epoxy monomers are synthesized from bisphenol A (BPA) [8,9]. Growing concerns over the environmental and health impacts of BPA, coupled with increasingly stringent regulatory requirements, have accelerated the development of bio-based epoxy monomers as alternatives to conventional BPA-based systems without compromising performance [1].

**Figure 1:**
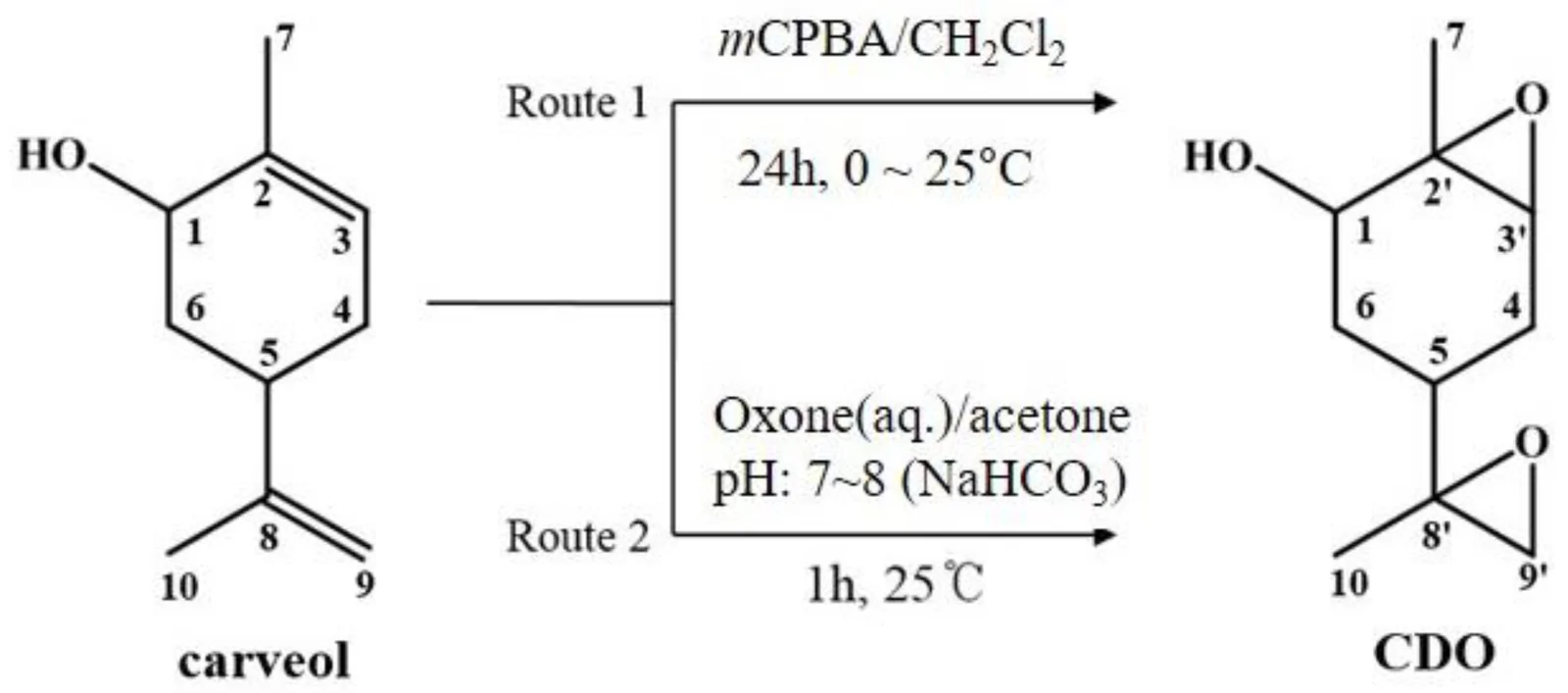
Synthesis of carveol dioxide (CDO) from carveol.

**Figure 2:**
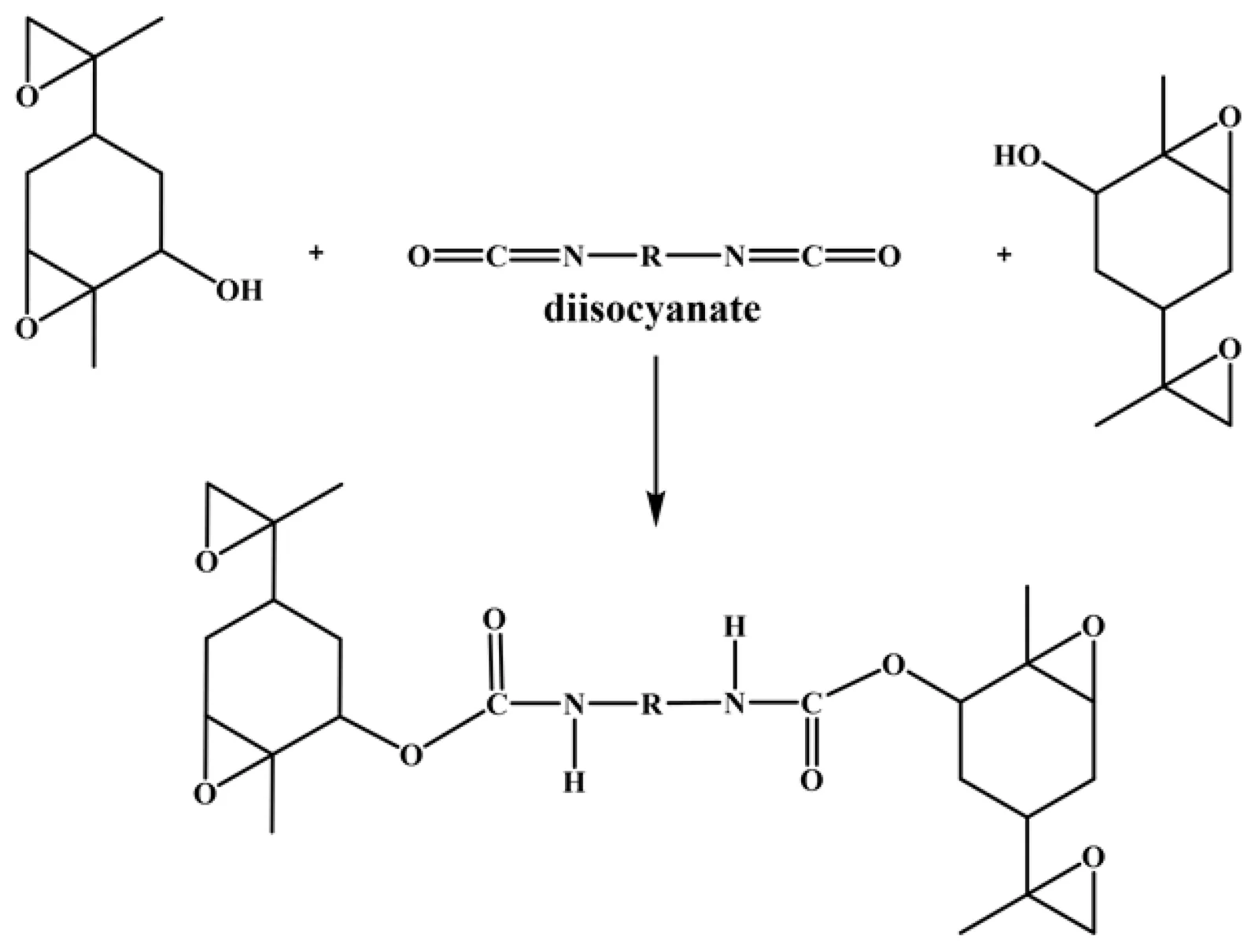
Formation of urethane linkages by reaction of CDO with diisocyanates.

**Figure 3:**
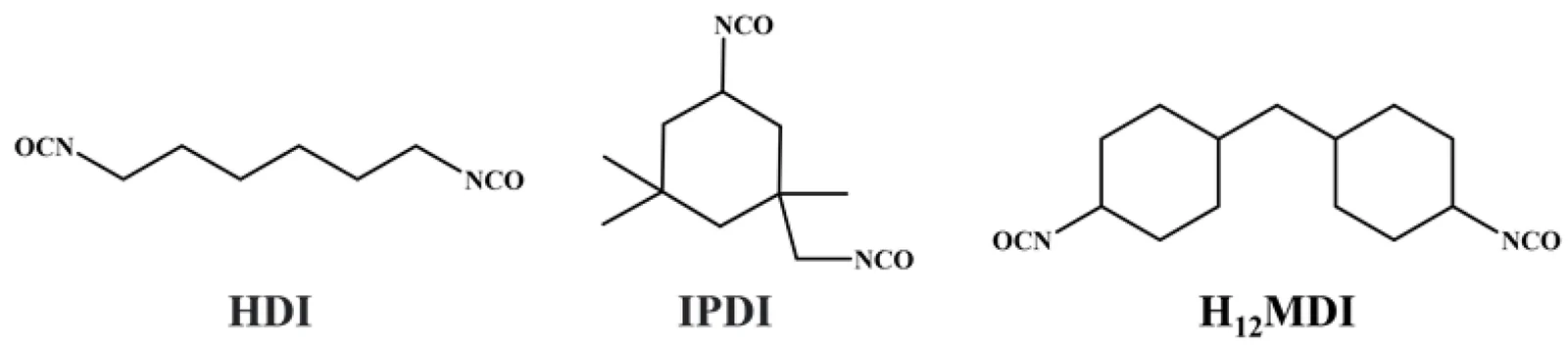
Chemical structures of HDI, IPDI, and H_12_MDI.

Terpenes and terpenoids, which are primarily obtained from essential oils, constitute an important class of renewable platform molecules for the synthesis of bio-based polymers [10,11,12,13]. Among them, cyclic monoterpenes are particularly attractive because their rigid ring structures enable the preparation of bio-based cycloaliphatic polymers with high *T*_g_ [13]. Furthermore, compared with conventional BPA-based aromatic epoxy monomers, cycloaliphatic epoxy monomers offer several unique advantages, including low dielectric constant, low dielectric loss, excellent weatherability, and superior UV resistance, making them attractive for high-performance electrical, electronic, coating, and adhesive applications [14,15,16,17]. Reflecting these advantages and the growing demand for renewable materials, the bio-based and cycloaliphatic epoxy markets are projected to grow at a compound annual growth rate (CAGR) of 6.66% during 2026–2031, substantially outpacing the projected CAGR of 3.53% for the overall epoxy resin market [7]. Limonene is one of the most abundant and extensively studied cyclic monoterpenes for the development of bio-based monomers [18,19,20,21,22]. Its epoxidation product, limonene oxide, has attracted considerable attention as a versatile bio-based monomer for the synthesis of polycarbonates [21,23], hydroxyurethanes [24,25], photopolymerized thermoset [26], epoxy-amine [27,28] and epoxy-anhydride thermosets [29]. Carveol, an alcohol derivative of limonene, is a renewable terpenoid consisting of a 2-methylcyclohex-2-en-1-ol ring bearing an isopropenyl substituent, thereby containing both an endocyclic and an exocyclic C=C double bond (Figure 1) [30,31,32]. Unlike limonene, carveol possesses a pendant secondary allylic hydroxyl group that provides an additional site for chemical modification, promotes intermolecular hydrogen bonding, and enables greater flexibility in network design. Despite extensive studies on limonene epoxidation, the epoxidation of carveol has received relatively limited attention, and, to the best of our knowledge, no previous studies have reported the curing behavior of epoxidized carveol or the properties of the resulting thermoset networks [33,34]. In this work, carveol was epoxidized to produce carveol dioxide (CDO), a difunctional cycloaliphatic epoxy monomer, which was subsequently cured with branched polyethyleneimine (BPEI). The resulting BPEI-cured CDO (BPEI–CDO) thermosets exhibited excellent thermomechanical properties and strong adhesion. Furthermore, the pendant hydroxyl group of CDO was reacted with aliphatic diisocyanates prior to epoxy–amine curing to introduce additional urethane crosslinks into the network (Figure 2 and Figure 3). Compared with the corresponding BPEI–CDO network without urethane modification, the resulting epoxy–urethane thermosets exhibited a higher crosslink density and a substantially increased *T*_g_. These findings demonstrate that carveol is a promising renewable platform molecule for the development of high-performance bio-based epoxy thermosets.

## 2. Materials and Methods

### 2.1. Materials

L-Carveol (95%, a mixture of cis and trans isomers; CAS No. 99-48-9), dibutyltin dilaurate (95%; CAS No. 77-58-7), sodium sulfite (Na_2_SO_3_, 98%; CAS No. 7757-83-7), Oxone^®^ (potassium peroxymonosulfate compound; CAS No. 70693-62-8), branched polyethyleneimine (BPEI, *M*_n_ = 600 g mol^−1^; CAS No. 25987-06-8), isophorone diisocyanate (IPDI; CAS No. 4098-71-9), and 4,4′-methylenebis(cyclohexyl isocyanate) (H_12_MDI, 90%; CAS No. 5124-30-1) were purchased from Sigma-Aldrich (St. Louis, MO, USA) and used as received. *m*-chloroperoxybenzoic acid (*m*CPBA, 70–75%; CAS No. 937-14-4) was purchased from Acros Organics (Geel, Belgium) and used as the oxidizing agent. Dichloromethane (DCM, 99.5%; CAS No. 75-09-2), methanol (95%; CAS No. 67-56-1), acetone (95%; CAS No. 67-64-1), sodium chloride (NaCl, 99.5%; CAS No. 7647-14-5), anhydrous magnesium sulfate (MgSO_4_, 99.5%; CAS No. 7487-88-9), 1,4-dioxane (99.5%; CAS No. 123-91-1), and sodium bicarbonate (NaHCO_3_, 99.5%; CAS No. 144-55-8) were purchased from Samchun Chemical Co. (Seoul, Republic of Korea). Hexamethylene diisocyanate (HDI; CAS No. 822-06-0) and ethyl acetate (99.8%; CAS No. 141-78-6) were purchased from Daejung Chemicals & Metals Co., Ltd. (Siheung, Republic of Korea). Sodium carbonate (Na_2_CO_3_; CAS No. 497-19-8) was purchased from Kanto Chemical Co., Inc. (Tokyo, Japan).

### 2.2. Synthesis of CDO Using mCPBA

The epoxidation of carveol can be achieved using peracids in organic solvents, such as *m*-chloroperbenzoic acid (*m*CPBA) in CH_2_Cl_2_, or dimethyldioxirane (DMDO), which is generated in situ from acetone and Oxone^®^ (potassium peroxymonosulfate). In this study, CDO was synthesized using both routes, and their epoxidation efficiencies were compared (Figure 1). Carveol was epoxidized using *m*CPBA according to a modified literature procedure [35,36]. L-Carveol (7.6115 g, 50 mmol) was dissolved in dichloromethane (DCM, 50 mL). A solution of *m*CPBA (150 mmol) in DCM (150 mL) was added dropwise to the L-carveol solution over 1 h at 0 °C. The mixture was then stirred for 16–24 h. Both steps were performed in air with mechanical stirring at 250 rpm. After completion of the reaction, 100 mL of 10 wt% aqueous Na_2_CO_3_ solution was added, and the mixture was stirred for 1 min to quench the excess oxidant. The resulting solids were removed by filtration. The organic phase was washed successively with 10 wt% aqueous Na_2_CO_3_ solution (200 mL × 3), saturated NaCl solution (200 mL), and deionized water (200 mL), dried over anhydrous MgSO_4_, and filtered. Finally, the solvent was removed under reduced pressure using a rotary evaporator (N-1200B, EYELA, Tokyo Rikakikai Co., Ltd., Tokyo, Japan) to afford CDO.

### 2.3. Synthesis of CDO Using Oxone/Acetone

Carveol was alternatively epoxidized using the Oxone/acetone system according to a modified literature procedure [19,22,36,37]. L-Carveol (7.6115 g, 50 mmol) was dissolved in acetone (200 mL), and NaHCO_3_ (20 g) was added to adjust the pH to 7–8. An aqueous Oxone solution (0.52 M, 300 mL) was added dropwise at a rate of 3 mL min^−1^ over 100 min at 25 °C, with mechanical stirring at 250 rpm in air, after which the reaction was continued for an additional 1 h. The pH was maintained at 7–8 throughout the reaction. After completion of the reaction, the mixture was filtered to remove insoluble solids, and ethyl acetate was added to the filtrate for liquid–liquid extraction. The organic phase was separated, dried over anhydrous MgSO_4_, and filtered. Finally, the solvent was removed under reduced pressure using a rotary evaporator to afford CDO.

### 2.4. Urethane Reaction of CDO with Diisocyanates

The urethane reaction of carveol dioxide (CDO) with diisocyanates was carried out according to the procedure shown in Figure 2. Dibutyltin dilaurate (1 wt% relative to CDO) was added to CDO (1.8423 g, 10 mmol). The mixture was heated to 50–70 °C under a nitrogen atmosphere, followed by the slow addition of 0.5–1 mmol of diisocyanate, corresponding to a diisocyanate-to-CDO molar ratio of 0.05–0.10 [38]. Aliphatic diisocyanates, including HDI, IPDI, and H_12_MDI, were selected because their degradation products are generally considered less toxic than those derived from aromatic diisocyanates. The chemical structures of the diisocyanates are shown in Figure 3. The reaction mixture was stirred with a magnetic stir bar at 150 rpm for 1 h, yielding urethane-containing epoxy monomers as light-yellow liquids.

### 2.5. Crosslinking Reaction of CDO and Urethane-Containing CDO with BPEI

**Figure 4:**
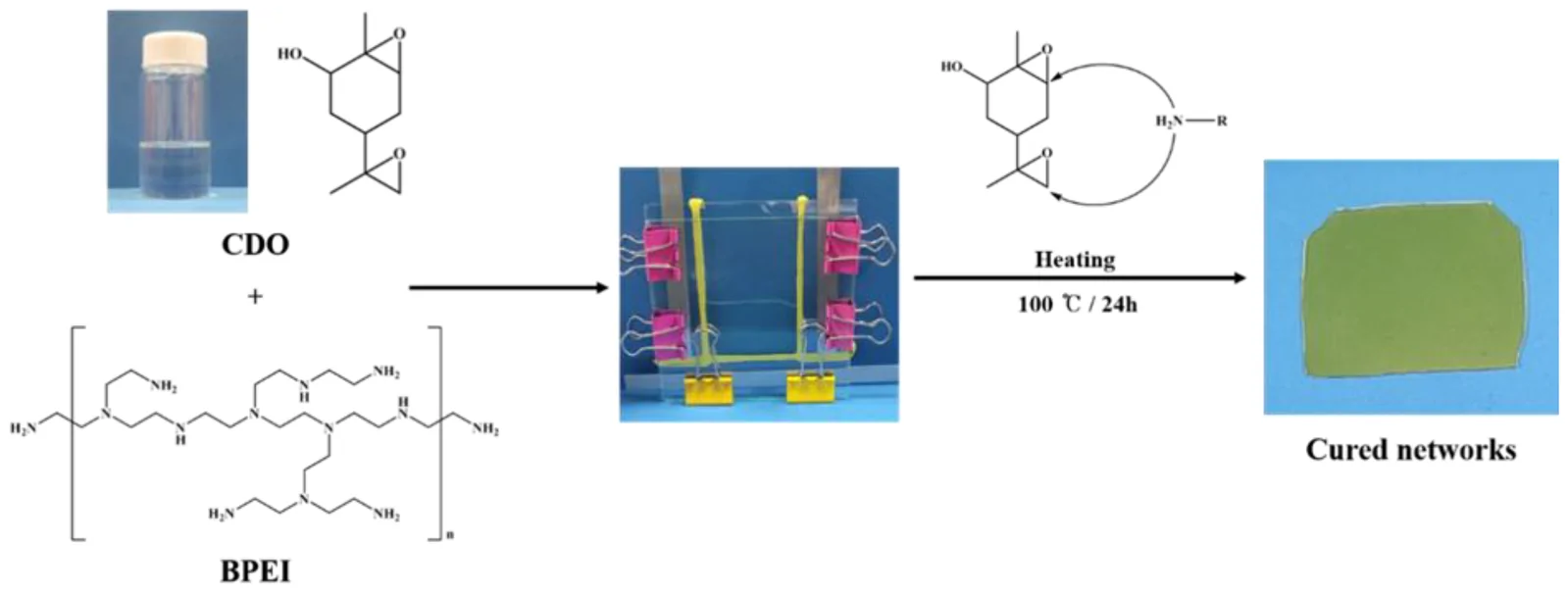
Curing process of CDO with BPEI.

A typical curing procedure using branched polyethyleneimine (BPEI) as the curing agent is illustrated in Figure 4. BPEI was mixed with CDO or urethane-modified CDO at various stoichiometric ratios. Based on quantitative NMR analysis, BPEI was estimated to contain, on average, approximately six primary amine groups and five secondary amine groups per molecule. The effective amine functionality of BPEI was calculated by treating each primary amine as difunctional and each secondary amine as monofunctional toward epoxy groups. Accordingly, amine-functionality-to-epoxy molar ratios of 0:1, 0.5:1, 1.0:1, and 1.5:1 were investigated. The mixtures were homogenized using a vortex mixer (VM-10, DAIHAN Scientific Co., Ltd., Wonju, Republic of Korea) for 1 min, followed by mechanical stirring for 2 min. The resulting homogeneous mixtures were poured into glass molds and cured at 100 °C for 24 h under ambient air. No residual curing exotherm was detected by differential scanning calorimetry, indicating that curing was essentially complete under the conditions employed.

### 2.6. Characterization

#### 2.6.1. Equivalent Epoxy Weight (EEW)

Epoxy equivalent weight (EEW) was determined as follows. Approximately 0.3 g of the oxidized carveol sample was dissolved in 100 mL of 1,4-dioxane, followed by the addition of 25 mL of 0.2 N HCl in 1,4-dioxane. The mixture was maintained at 25 °C for 30 min and subsequently titrated with 0.1 N NaOH in methanol using cresol red as the indicator until a violet endpoint was observed. A blank titration was performed under identical conditions. The EEW was calculated using Equation (1).

(1) $$\mathrm{EEW}(g/\mathrm{eq})=\frac{w\;\times \;1000\;\times \;f}{N\;\times \;(V_{1}-V_{2})}$$

where w is the weight of the sample, N is the normality of the NaOH solution (0.1 N), V_1_ and V_2_ denote the volume of the blank and sample, respectively, and f represents the correction factor of the NaOH solution [39].

#### 2.6.2. Fourier Transform Infrared (FT–IR) Spectroscopy

Changes in the chemical structures of the reactants during each synthesis step were monitored using Fourier transform infrared (FT-IR) spectroscopy. FT-IR analysis was conducted utilizing a Bruker/Tensor27 spectrometer. Spectra were acquired from the samples deposited on the attenuated total reflection (ATR) plate at a resolution of 4 cm^−1^ with 16 scans co-added for each measurement.

#### 2.6.3. Nuclear Magnetic Resonance (NMR)

Proton nuclear magnetic resonance (^1^H–NMR) analyses of the samples were recorded in deuterated chloroform (CDCl_3_) using an AvanceIII-500 MHz Spectrometer (Bruker, Germany (Bruker BioSpin, Rheinstetten, Germany)) at 25 °C [35,40].

#### 2.6.4. Dynamic Mechanical Analysis

Dynamic mechanical analysis (DMA) was performed using a DMA/SDTA861e instrument (Mettler Toledo, Greifensee, Switzerland). The cured samples were cut into rectangular specimens with dimensions of 5 × 25 × 1 mm^3^. The specimens were tested in uniaxial tensile mode at a frequency of 1 Hz over a temperature range of −40 to 200 °C at a heating rate of 3 °C/min. *T*_g_ was determined from the peak maximum of the tan δ curve. At temperatures above *T*_g_, the thermoset enters the rubbery plateau region, where the crosslink density (ν) can be estimated using the rubber elasticity theory as follows [27,28]:

(2) $$E^{\prime }=\emptyset RT\nu$$

where *E′* is the storage modulus in the rubbery plateau region, *R* is the universal gas constant, *T* is the absolute temperature, ν is the crosslink density, and φ is a front factor, which was assumed to be unity in this study. The reported *E′*, *T*_g_ and *ν* values represent the averages of five independent DMA measurements.

#### 2.6.5. Gel Content

The gel content was determined to evaluate the degree of crosslinking of the cured samples. Approximately 30 mg of each cured sample was immersed in methanol for 72 h at room temperature to extract the soluble fraction. After immersion, the remaining insoluble samples were dried in a ventilated oven at 70 °C for 24 h to constant weight. The gel content (GC) was calculated using Equation (5) [27,41]:

(3) $$\mathrm{GC}\left(\%\right)=\;\frac{m_{2}}{m_{1}}\times 100$$

where $m_{2}$ denotes the mass of the immersed samples after drying in the oven and $m_{1}$ represents the initial mass of the samples.

#### 2.6.6. Adhesion Test

The adhesive performance of CDO-based formulations was evaluated by lap-shear testing using a universal testing machine (Instron 5569, Instron, Norwood, MA, USA) equipped with a 50 kN load cell. Stainless-steel adherends (12.5 × 101.6 × 1.5 mm^3^) were prepared in accordance with ASTM D1002 [42], with an overlap area of 12.5 × 25 mm^2^. The uncured CDO/BPEI mixture was applied to the overlap region, after which the adherends were assembled, secured with binder clips, and cured in an oven at 100 °C for 24 h. The lap-shear strength was calculated by dividing the maximum load at failure by the bonded overlap area. Five specimens were tested for each adhesive formulation, and the reported values represent the mean of five measurements.

## 3. Results and Discussion

### 3.1. Epoxidation of Carveol Using mCPBA

**Figure 5:**
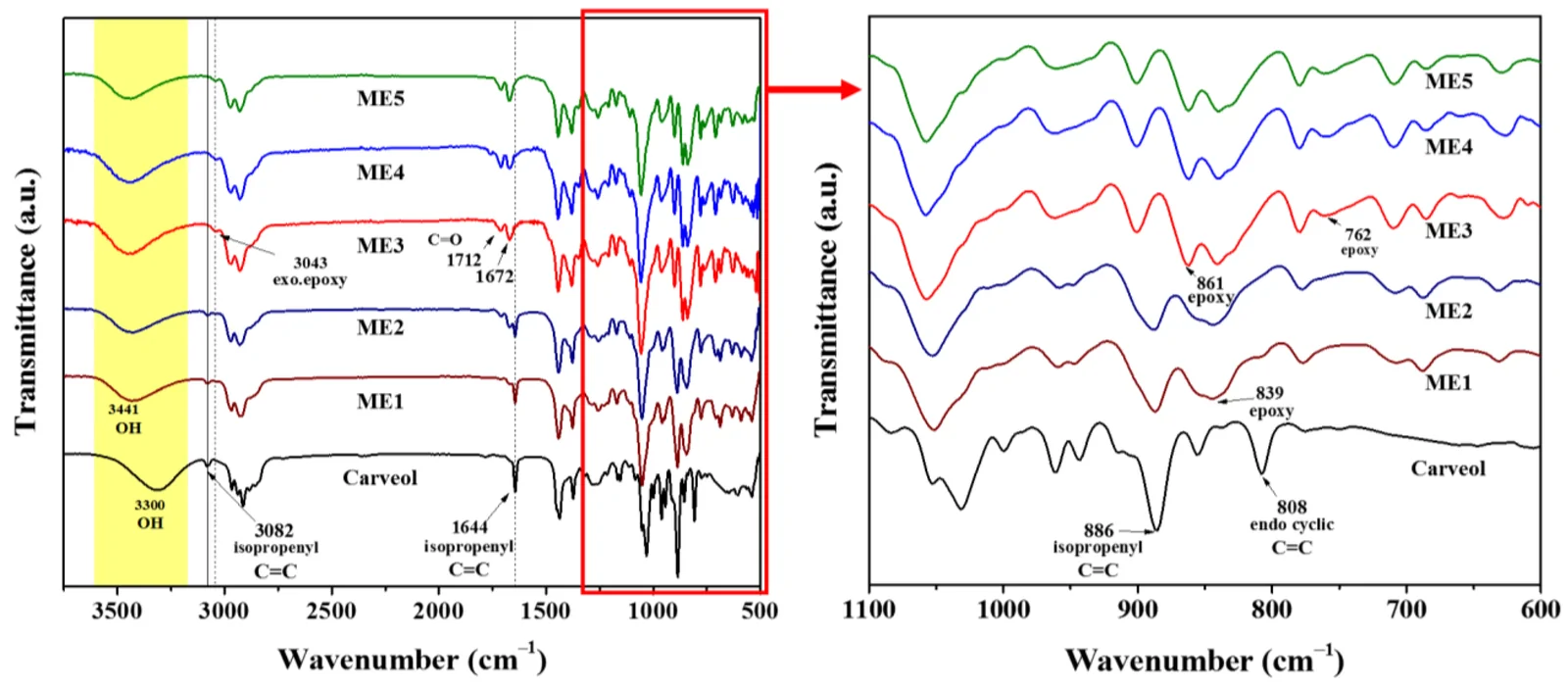
FT-IR spectra of the starting material and *m*CPBA-derived epoxidation products. The FT-IR spectrum of ME6 was nearly identical to that of ME5.

Carveol is an unsaturated alcohol containing two double bonds: an exocyclic isopropenyl C=C bond and an endocyclic C=C bond within the cyclohexene ring. Both C=C bonds of carveol were epoxidized using *m*CPBA as the oxidizing agent to produce CDO. To optimize the synthesis of CDO, the effects of reaction temperature, reaction time, and the *m*CPBA-to-carveol molar ratio were systematically investigated, as summarized in Table 1. FT-IR spectroscopy was used to monitor carveol epoxidation under different reaction conditions. In the FT-IR spectrum of carveol (Figure 5), the absorption bands at 1644 and 3082 cm^−1^ were assigned to the stretching vibration of the exocyclic isopropenyl C=C bond, whereas the bands at 886 and 808 cm^−1^ were attributed to the out-of-plane bending vibrations of the exocyclic and endocyclic double bonds, respectively [17,18]. These bands decreased markedly after epoxidation and completely disappeared in ME3–ME5. In contrast, the epoxidized samples exhibited characteristic absorption bands at 762, 839, and 861 cm^−1^, confirming the formation of epoxide groups [17,26,28]. In ME1, the residual bands at 3082, 1644 and 886 cm^−1^ remained, whereas the band at 808 cm^−1^ nearly disappeared, indicating incomplete epoxidation and higher reactivity of the endocyclic double bond relative to the exocyclic double bond. Increasing the reaction temperature from 0 to 25 °C (ME2) was insufficient to completely eliminate the residual C=C-related bands, whereas increasing the *m*CPBA-to-carveol molar ratio from 2:1 to 3:1 (ME3) led to their disappearance, indicating nearly complete epoxidation. The broad O–H stretching band of carveol around 3300 cm^−1^ shifted to a higher wavenumber after epoxidation, suggesting changes in hydrogen-bonding interactions due to altered local polarity, while its persistence indicated that most hydroxyl groups were retained. However, new bands at 1672 and 1712 cm^−1^ appeared in ME2–ME5 and were assigned to carbonyl groups [35], indicating partial oxidation of the secondary hydroxyl groups by *m*CPBA as a side reaction.

**Figure 6:**
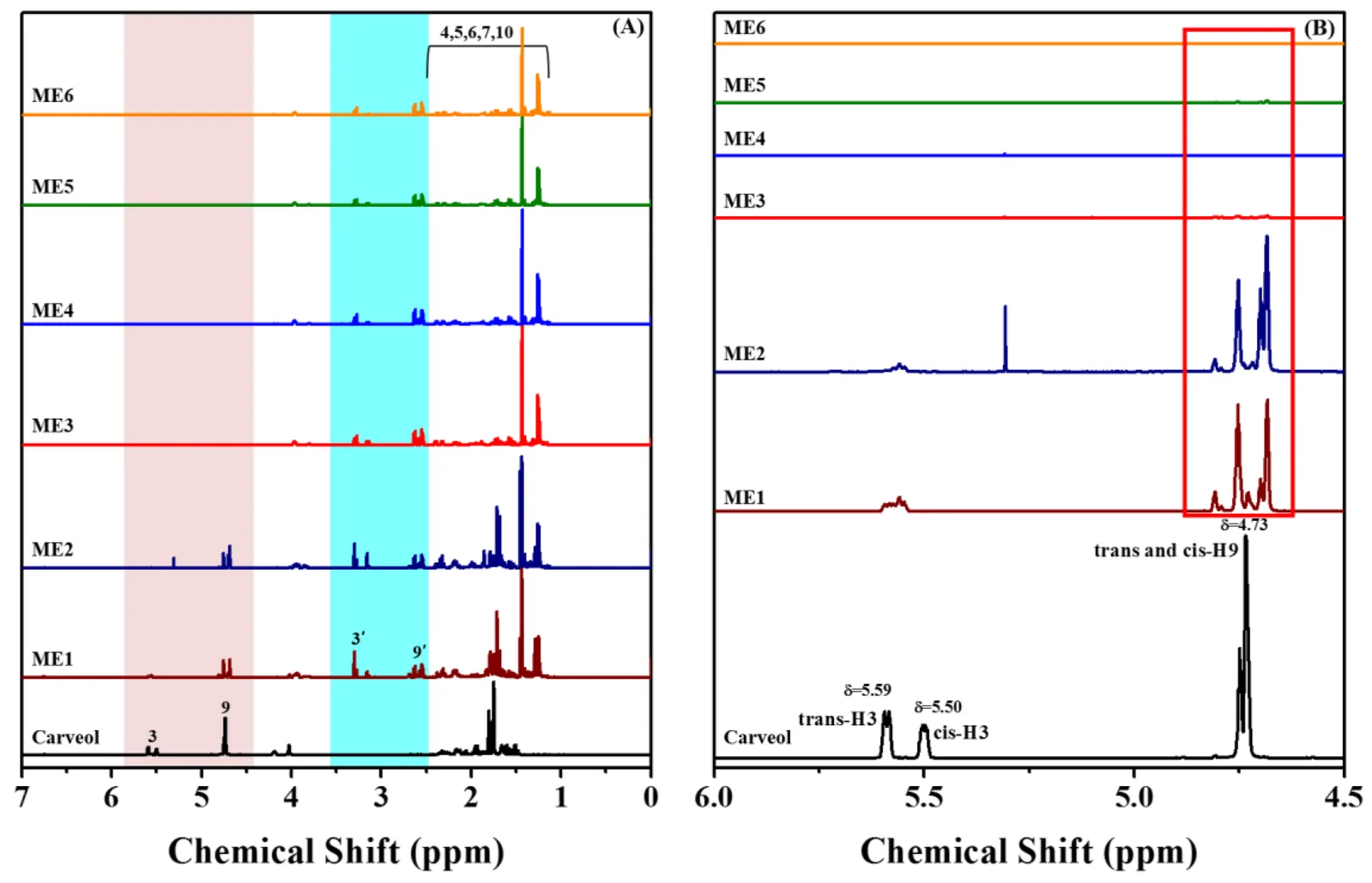
^1^H-NMR spectra of carveol and the epoxidation products obtained using *m*CPBA: (**A**) full spectra over δH = 0–7 ppm and (**B**) expanded olefinic region at δH = 4.5–6.0 ppm.

To further characterize the structural changes associated with epoxidation, the oxidized carveol samples prepared using *m*CPBA were analyzed by ^1^H NMR spectroscopy. In the spectrum of the starting carveol (Figure 6), the signals at δH = 4.5–6.0 ppm were assigned to the olefinic protons of the exocyclic C=C bond (H9) and the endocyclic C=C bond (H3), as indicated in Figure 1. These signals exhibited complex splitting patterns associated with the presence of cis/trans isomers. No residual olefinic signals were detected in ME3–ME6, whereas such signals remained in ME1 and ME2, indicating incomplete consumption of the C=C bonds, consistent with the FT-IR results. The persistence of the H9 signals suggests slower consumption of the exocyclic C=C bond; however, direct comparison of the H9 and H3 signal intensities requires caution because they correspond to two and one protons, respectively. Preferential epoxidation of the endocyclic C=C bond is consistent with previous reports that trisubstituted endocyclic C=C bonds undergo epoxidation approximately seven times faster than disubstituted exocyclic C=C bonds [19].

New signals appeared at δH = 2.5–3.5 ppm and were assigned to the epoxide-ring protons (H3′ and H9′; Figure 6A). These signals were more prominent in ME3–ME6, supporting the formation of epoxide groups. Together, the disappearance of detectable olefinic signals and the appearance of epoxide resonances indicate that an *m*CPBA-to-carveol molar ratio of 3:1 was effective for epoxidation under the conditions investigated.

The EEW decreased from 168 and 161 g eq^−1^ for ME1 and ME2, respectively, to approximately 126.7 g eq^−1^ for ME3–ME6, indicating an increase in epoxy group content per unit mass, consistent with the qualitative NMR observations. Nevertheless, this value remained above the theoretical EEW of pure CDO (92.1 g eq^−1^), indicating a lower epoxy group content than expected for the pure diepoxide. This discrepancy may reflect the presence of residual components or by-products with lower epoxy functionality, potentially including products of epoxide ring-opening reactions, although their contributions could not be established from the present data. Accordingly, the disappearance of detectable olefinic resonances supports C=C consumption but does not establish quantitative formation of CDO or a 100% epoxide yield.

### 3.2. Epoxidation of Carveol Using Oxone/Acetone

**Figure 7:**
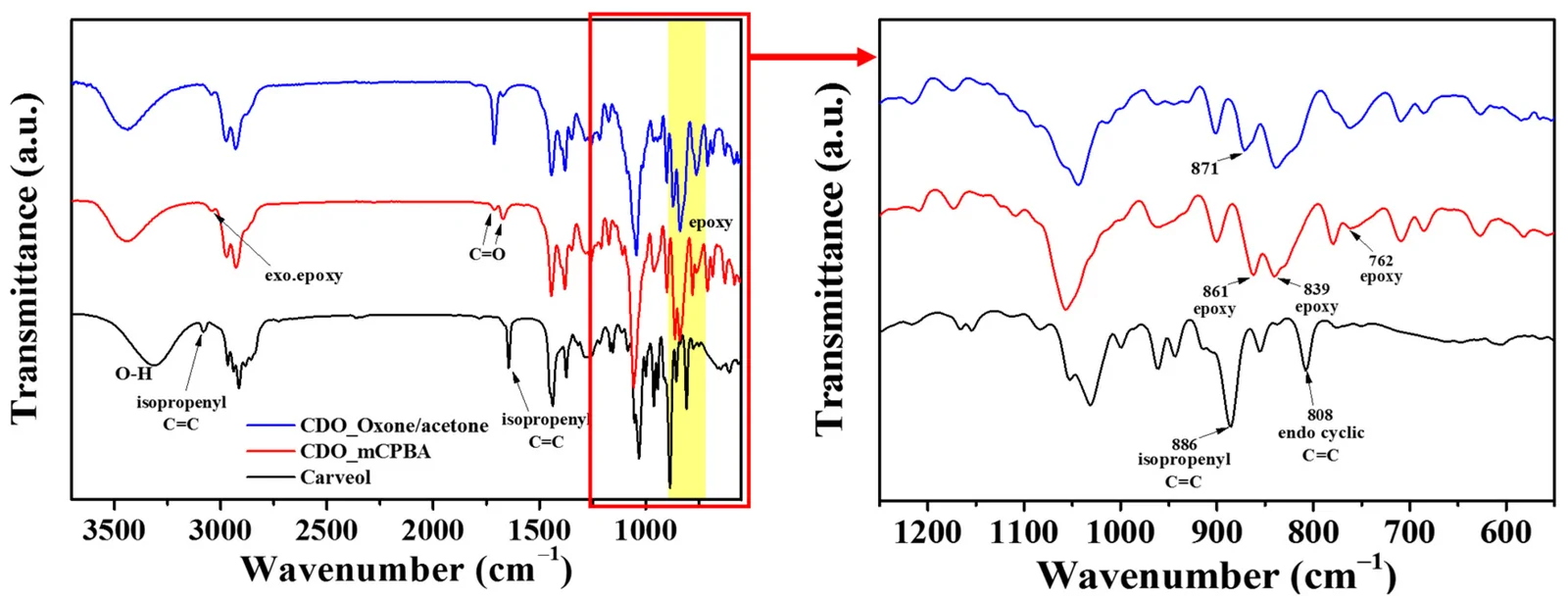
FT-IR spectra of starting material and products of epoxidation using *m*CPBA (ME5) or Oxone/acetone (OE2).

CDO was also synthesized using an alternative Oxone/acetone epoxidation method under various reaction conditions. An Oxone-to-carveol molar ratio of 3:1 at 25 °C for 1 h afforded high conversion and CDO yield (Table 2). Under these conditions, complete epoxidation was achieved within 1 h, substantially reducing the reaction time compared with the 16–24 h required for *m*CPBA-mediated epoxidation. As shown in the FT-IR spectrum of OE2 (Figure 7), Oxone/acetone epoxidation, similar to *m*CPBA-mediated oxidation, resulted in the disappearance of C=C-related bands and the appearance of characteristic epoxy bands, confirming successful epoxidation by both methods. Notably, OE2 exhibited a more intense carbonyl (C=O) stretching band at 1712 cm^−1^ than ME5, suggesting more extensive oxidation of the hydroxyl groups by the Oxone/acetone system. The O–H stretching band and the epoxide absorption band at 861 cm^−1^ were slightly blue-shifted in the Oxone-oxidized product, suggesting changes in the local chemical environments of the hydroxyl and epoxide groups. These shifts may be associated with altered hydrogen-bonding interactions arising from the greater abundance of carbonyl-containing by-products in the Oxone-oxidized sample.

**Figure 8:**
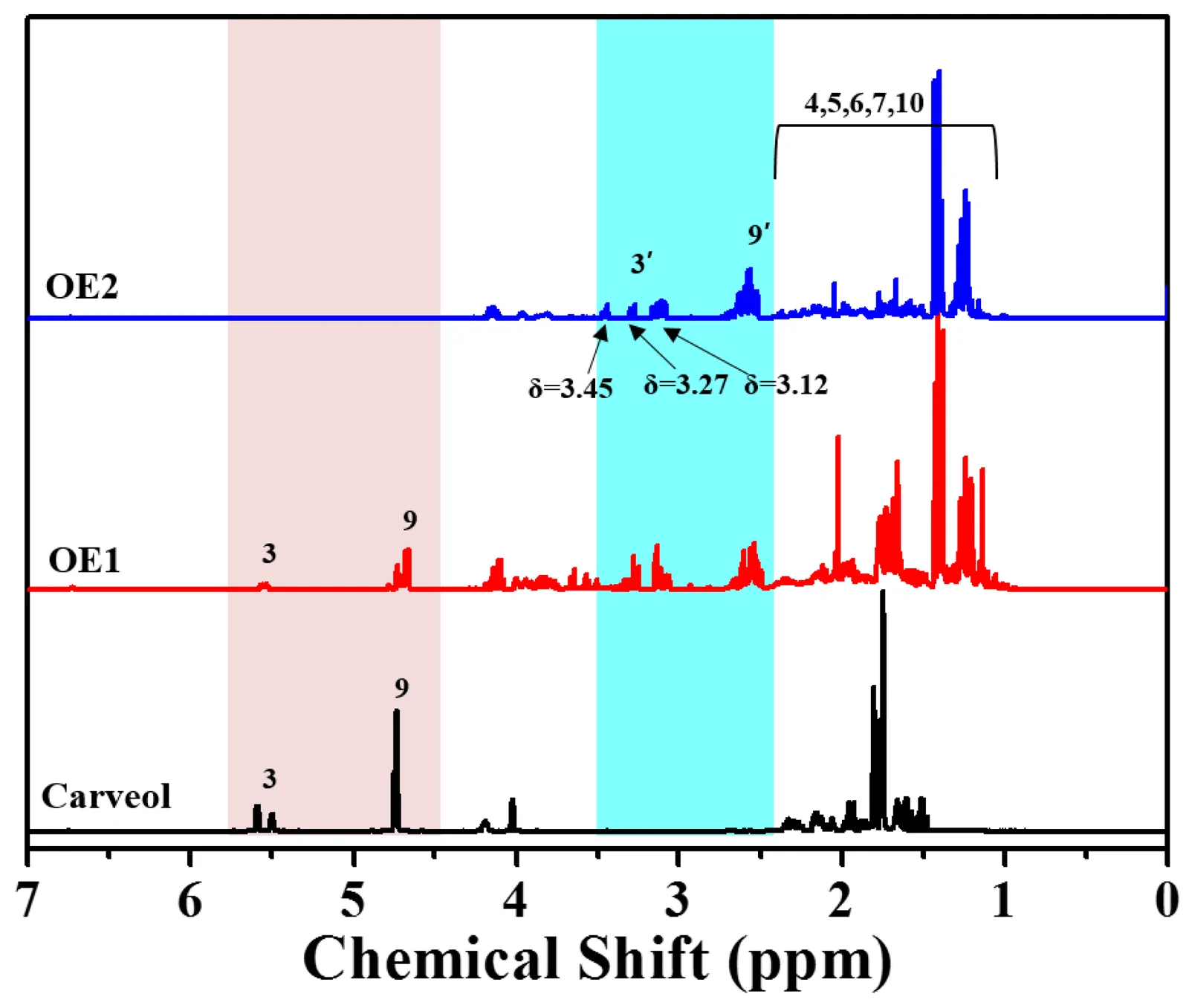
^1^H-NMR spectra of the starting material and epoxidation products obtained using Oxone/acetone.

The ^1^H NMR spectra of carveol before and after Oxone/acetone epoxidation at 0 and 25 °C are shown in Figure 8. At 0 °C, the H9 signals remained markedly more intense than the H3 signals, indicating incomplete epoxidation and preferential retention of the exocyclic C=C bond (OE1), consistent with the behavior observed for *m*CPBA-mediated epoxidation. By contrast, no olefinic proton signals were detected after reaction at 25 °C, confirming complete epoxidation of carveol under these conditions. Correspondingly, new signals appeared at δH = 2.5–3.5 ppm, which were assigned to the epoxide-ring protons (H3′ and H9′), consistent with those observed for CDO prepared using *m*CPBA. Unlike *m*CPBA, which is hazardous, generates stoichiometric amounts of *m*-chlorobenzoic acid waste and often requires chlorinated solvents for purification, the Oxone/acetone system avoids metal catalysts and chlorinated solvents, simplifies product isolation, and provides a more environmentally benign route to CDO [29,37].

### 3.3. Urethane Reaction of CDO with Diisocyanates

**Figure 9:**
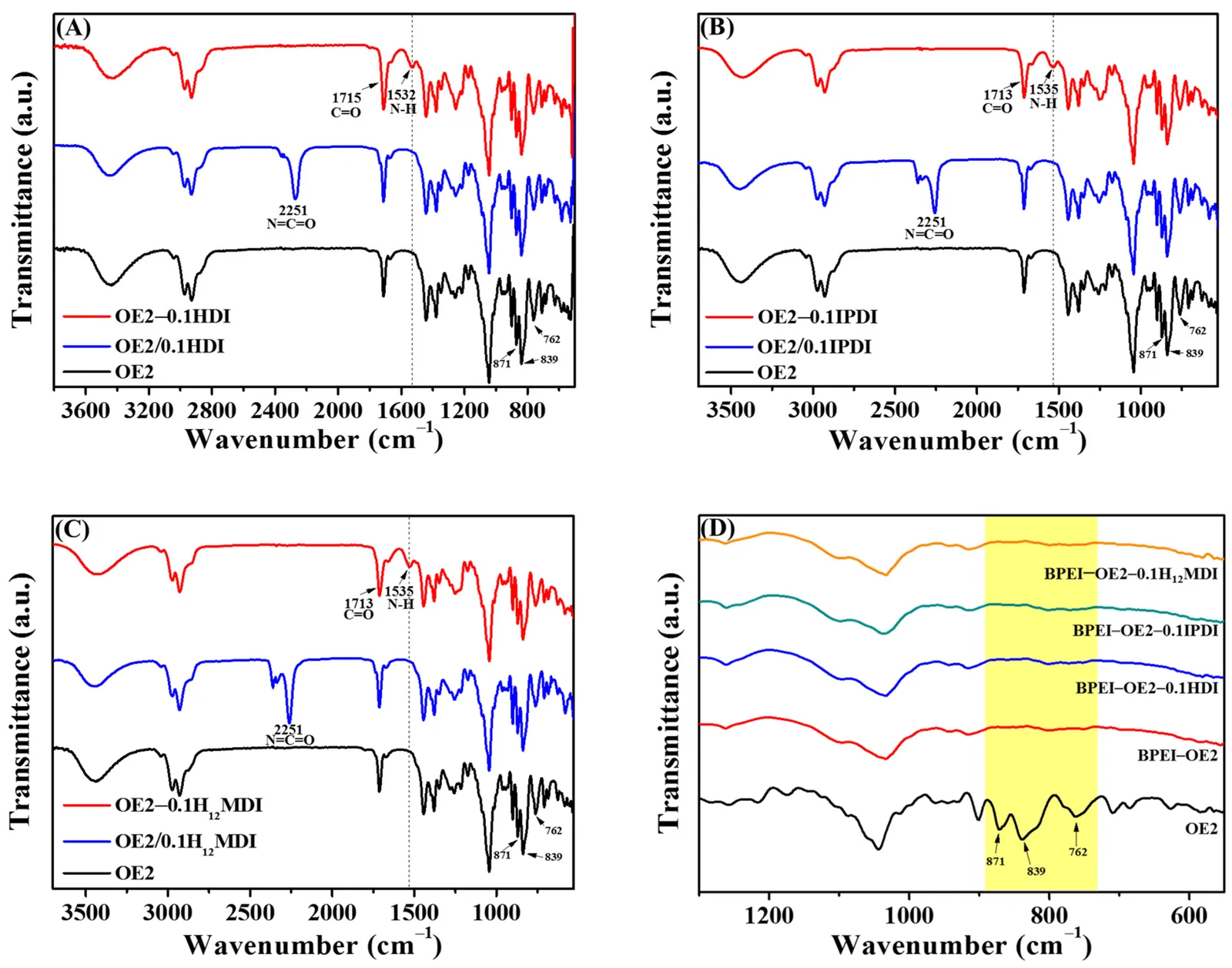
(**A**–**C**); Comparison of the FT-IR spectra of CDO before and after reaction with diisocyanates. OE2: neat CDO; OE2/0.1HDI, OE2/0.1IPDI and OE2/0.1H_12_MDI: mixtures of CDO and diisocyanates prior to urethane formation at an epoxy-to-isocyanate molar ratio of 1:0.1; OE2−0.1HDI, OE2−0.1IPDI and OE2−0.1H_12_MDI; corresponding products after urethane formation. (**D**) Comparison of the FT-IR spectra of neat CDO monomer (OE2), BPEI-cured CDO (BPEI-OE2), and BPEI-cured urethane-containing CDO samples (BPEI-OE2-0.1HDI, BPEI-OE2-0.1IPDI, and BPEI-OE2-0.1H12MDI).

FT-IR spectroscopy was used to monitor the reaction of CDO with various diisocyanates (Figure 9A–C). Before reaction, the CDO/diisocyanate mixtures exhibited a characteristic absorption band at approximately 2251 cm^−1^, assigned to the N=C=O stretching vibration of the isocyanate groups. The complete disappearance of this band after reaction confirmed consumption of the isocyanate groups. The resulting products exhibited urethane-associated bands at 1713–1715 cm^−1^ and 1532–1535 cm^−1^, attributed to C=O stretching and N–H bending vibrations, respectively [43,44]. Although the urethane C=O band overlaps with the carbonyl absorption arising from by-products formed during Oxone-mediated epoxidation, the emergence of the N–H bending band at 1532–1535 cm^−1^ supports successful urethane formation. A relevant competing pathway is oxazolidinone formation through isocyanate–epoxide cycloaddition [45]. However, the characteristic epoxide absorption bands remained essentially unchanged, and no new band attributable to oxazolidinone C=O stretching was observed near 1750 cm^−1^. Together with the evidence of urethane formation, these observations support the selective reaction of isocyanate groups with the pendant hydroxyl groups of CDO, with no detectable isocyanate–epoxide reaction under the conditions employed. The epoxide functionality was therefore preserved for subsequent amine curing.

### 3.4. Thermoset Curing of CDO and Urethane-Modified CDO, and Thermomechanical and Adhesive Properties of the Resulting Thermosets

CDO and urethane-modified CDO were cured with BPEI to form cross-linked thermoset networks. In this system, both the inherent secondary hydroxyl group of CDO and the hydroxyl groups generated during curing may promote intermolecular hydrogen bonding and facilitate the epoxy–amine reaction through an autocatalytic effect. Although these hydroxyl groups can also open epoxide rings to form ether linkages, this slower reaction is expected to contribute only marginally as long as sufficient active amine hydrogens remain available. The cured samples swelled extensively in methanol, while the soluble fraction was extracted during the swelling test. Their swelling and dissolution behavior was evaluated to assess the degree of crosslinking and network integrity. As summarized in Table 3, the thermoset prepared at an amine-functionality-to-epoxy molar ratio of 1:1 exhibited the highest gel content, indicating the formation of the most highly crosslinked network. This result is consistent with a previous study on the curing of limonene dioxide with BPEI [28]. Figure 9D shows the FT-IR spectra of CDO before and after curing with BPEI. The characteristic epoxide absorption bands at 762, 839, and 871 cm^−1^ disappeared after curing, indicating amine-induced ring opening of the epoxide groups during thermoset formation. Previous studies on LDO have shown that the exocyclic epoxide readily undergoes amine ring opening, whereas the reactivity of the endocyclic epoxide is strongly dependent on cis/trans stereochemistry. Amine-induced ring opening of the endocyclic epoxide has been reported to occur predominantly for the trans isomers because reaction of the cis isomers requires a higher-energy, boat-like transition state, consistent with the Fürst–Plattner rule [20,21,27,28]. Although CDO contains an additional hydroxyl substituent that introduces an extra stereogenic center and increases stereoisomeric complexity compared with LDO, the hydroxyl group is remote from the endocyclic epoxide. Therefore, CDO may exhibit stereochemical behavior similar to LDO, with isomers having a trans relationship between the methyl and epoxidized isopropenyl substituents preferentially participating in amine curing, in accordance with the Fürst–Plattner rule.

**Figure 10:**
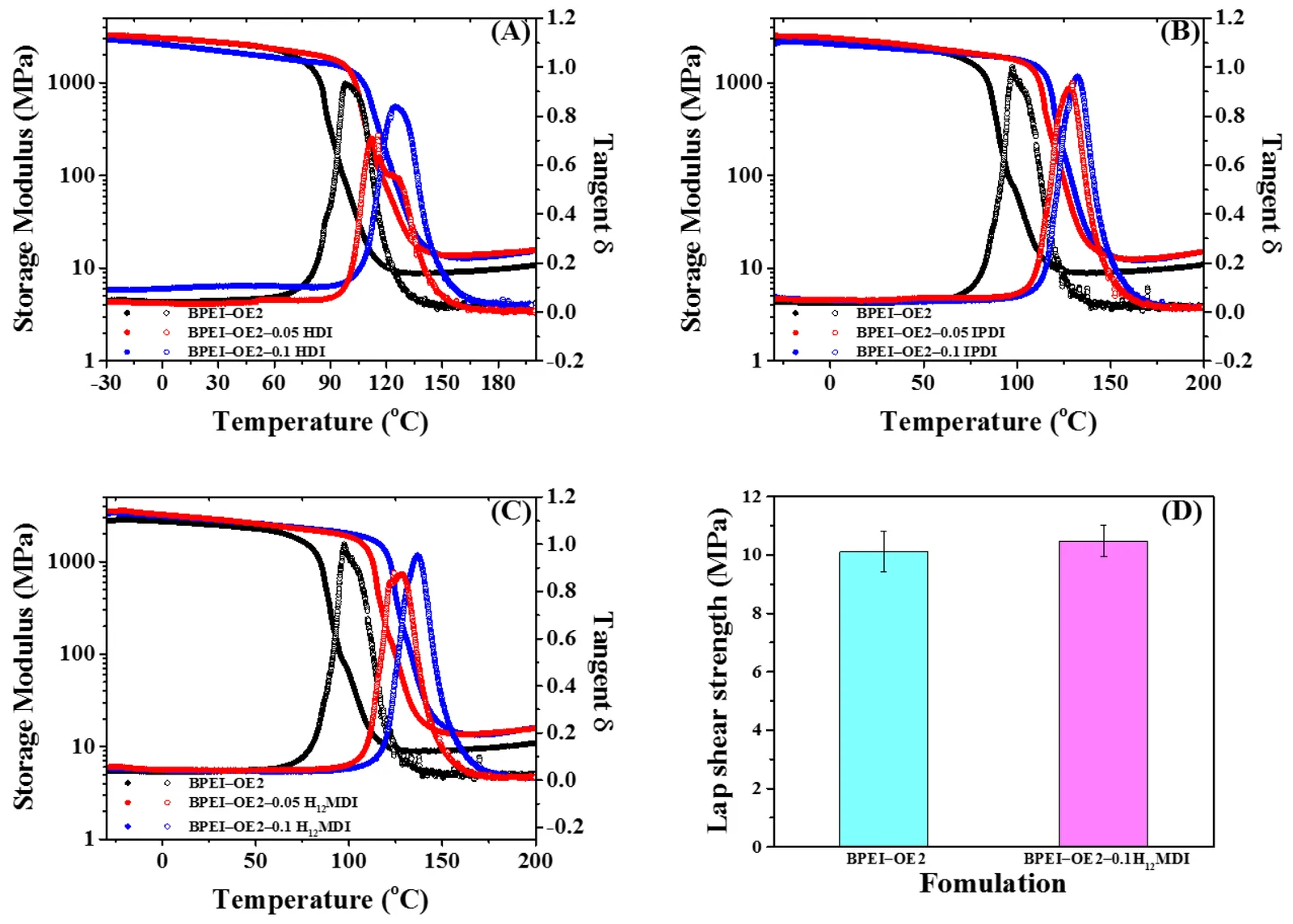
(**A**–**C**) Effects of urethane modification on the storage modulus and loss tangent (tan δ) of BPEI–CDO (OE2) networks measured by DMA. The amine-functionality-to-epoxy molar ratio was fixed at 1:1. The diisocyanates used for urethane modification were (**A**) HDI, (**B**) IPDI, and (**C**) H_12_MDI. (**D**) Lap-shear strength of BPEI−OE2 and BPEI−OE2−0.1H_12_MDI on stainless steel substrates.

The thermomechanical behavior of the BPEI-cured CDO thermosets was investigated by dynamic mechanical analysis (DMA). As shown in Figure 10A−C, the storage modulus (E′), loss modulus (E″), and tan δ were monitored as a function of temperature for thermosets derived from CDO and urethane-modified CDO. The *T*_g_ was defined as the temperature corresponding to the maximum of the tan δ curve. The influence of urethane incorporation on the gel content and thermomechanical properties of the cured networks is summarized in Table 4.

The BPEI–CDO network without urethane linkages exhibited a storage modulus (E′) of 2.49 GPa at 30 °C and a *T*_g_ of 97.4 °C, markedly higher than the corresponding values of approximately 1 GPa and 70 °C reported for BPEI-cured LDO [28]. The superior thermomechanical properties of the BPEI–CDO network may be attributed, at least in part, to intermolecular hydrogen-bonding interactions involving the hydroxyl groups of CDO. The incorporation of urethane linkages into the BPEI–CDO thermosets resulted in only a modest increase in storage modulus but substantially increased the apparent crosslink density estimated from the rubbery storage modulus and *T*_g_. In particular, *T*_g_ increased from 97.4 to 137 °C, representing a substantial enhancement over that of the BPEI–CDO network and, to the best of our knowledge, the highest *T*_g_ reported to date for a terpene-based epoxy thermoset. This pronounced enhancement is attributed primarily to the additional crosslinking points introduced by the urethane linkages, which increase network connectivity and restrict segmental mobility within the epoxy–amine network. The rigidity of the incorporated diisocyanate segments and hydrogen-bonding interactions involving the urethane groups may also contribute to the increased Tg, although their individual contributions could not be distinguished from the effect of increased crosslink density. Finally, lap-shear tests were conducted to evaluate the adhesive performance of the BPEI-cured CDO thermosets. The BPEI-cured neat CDO network exhibited a lap-shear strength of 10.1 MPa, while the incorporation of urethane linkages produced only a modest further improvement (Figure 10D), in contrast to the pronounced increase in *T*_g_ observed upon urethane modification. The relatively high adhesive strength of the BPEI–CDO thermosets is likely associated with the high concentration of polar hydroxyl groups arising from both the intrinsic secondary hydroxyl functionality of CDO and the additional hydroxyl groups generated through epoxy ring-opening during amine curing. Notably, the lap-shear strength of the BPEI–CDO thermosets exceeds the values reported for some conventional BPA-based epoxy adhesives and recently developed bio-based adhesives (4–7 MPa) [46,47,48,49].

## 4. Conclusions

This study demonstrates the potential of carveol as a renewable precursor for cycloaliphatic epoxy thermosets, with its pendant secondary hydroxyl group providing an additional site for network modification. Curing carveol dioxide (CDO) with branched polyethyleneimine yielded a thermoset with a storage modulus of 2.49 GPa at 30 °C, a glass transition temperature (*T*g) of 97.4 °C, and a stainless-steel lap-shear strength of 10.1 MPa. Subsequent network design through hydroxyl-mediated urethane formation prior to epoxy–amine curing increased the crosslink density and raised *T*g to 137 °C. The principal contribution of this work is the use of the intrinsic hydroxyl functionality of CDO to extend the network-design possibilities of terpene-derived epoxies. This approach establishes a route to tailoring the thermal performance of bio-based epoxy thermosets and supports their further development for adhesive applications.

## Figures and Tables

**Table 1:** Oxidized carveols synthesized using *m*CPBA under different reaction conditions.

Sample	Mole Ratio of Reactants	Reaction Time	Reaction Temperature	EEW
	mCPBA: Carveol	(h)	(°C)	(g/eq)
ME1	2:1	16	0	168.78
ME2	2:1	16	25	161.41
ME3	3/1	16	0	128.82
ME4	3:1	16	25	130.74
ME5	3:1	24	0	126.77
ME6	3:1	24	25	134.74

**Table 2:** Oxidized carveols synthesized using Oxone/acetone under different reaction conditions.

Sample	Oxone/Carveol Molar Ratio	Reaction Time (h)	Reaction Temperature (°C)	EEW (g/eq)
OE1	3/1	1	0	151.48
OE2	3/1	1	25	123.56

**Table 3:** Gel contents of BPEI–CDO networks at various amine-functionality-to-epoxy molar ratios.

Amine-Functionality-to-Epoxy Molar Ratios	Gel Content (%)
0:1	-
0.5:1	44.1
1:1	83.9
1.5:1	81.7

**Table 4:** Effect of diisocyanates on the properties of BPEI–CDO thermosets.

Amine-Functionality-to-Epoxy Molar Ratios	Diisocyanate Content (Molar Ratio Relative to CDO)			Gel Content (%)	E′ at 30 °C (GPa)	$T_{g}$ (°C)	E′ at $T_{g}$ + 40 °C (Mpa)	*ρ* (mol/L)
	HDI	IPDI	H_12_MDI					
1:1				83.9	2.49	97.4	8.85	2.59
1:1	0.05			92.7	2.76	112.35	13.87	3.92
1:1	0.1			94.1	2.22	121.4	12.91	3.94
1:1		0.05		81.4	2.73	128.2	12.67	3.45
1:1		0.1		92.9	2.40	132.35	12.46	3.36
1:1			0.05	84.5	2.86	128.85	13.75	3.74
1:1			0.1	95.8	2.82	136.55	13.65	3.65

## Data Availability

The original contributions presented in the study are included in the article, further inquiries can be directed to the corresponding author.
